# The effect of foreign language in fear acquisition

**DOI:** 10.1038/s41598-018-19352-8

**Published:** 2018-01-18

**Authors:** Azucena García-Palacios, Albert Costa, Diana Castilla, Eva del Río, Aina Casaponsa, Jon Andoni Duñabeitia

**Affiliations:** 1 Department of Basic and Clinical Psychology and Psychobiology, Jaume I University, Castellón, Spain; 2 Ciber. Fisiopatología Obesidad y Nutrición, (CIBERObn), Instituto de Salud Carlos III, Madrid, Spain; 3 Center for Brain and Cognition, Universitat Pompeu Fabra, Barcelona, Spain; 4 Institució Catalana de Recerca i Estudis Avançats (ICREA), Barcelona, Spain; 5 Department of Linguistics and English Language, Lancaster University, Lancaster, United Kingdom; 6 Facultad de Lenguas y Educación, Universidad Nebrija, Madrid, Spain; 7 Basque Center on Cognition, Brain and Language (BCBL), Donostia, Spain

## Abstract

Emotions are at the core of human nature. There is evidence that emotional reactivity in foreign languages compared to native languages is reduced. We explore whether this emotional distance could modulate fear conditioning, an essential mechanism for the understanding and treatment of anxiety disorders. A group of participants was verbally informed (either in a foreign or in a native language) that two different stimuli could be either cueing the potential presence of a threat stimulus or its absence. We registered pupil size and electrodermal activity and calculated the difference in psychophysiological responses to conditioned and to unconditioned stimuli. Our findings provided evidence that verbal conditioning processes are affected by language context in this paradigm. We report the first experimental evidence regarding how the use of a foreign language may reduce fear conditioning. This observation opens the avenue to the potential use of a foreign language in clinical contexts.

## Introduction

About 1800 years ago, a tombstone for Regina was set near the Hadrian’s Wall in England. Regina was a slave who was freed and married Barates, a man from Palmyra. Barates erected a monument with a Latin inscription dedicated to her: “*To the spirits of the departed and to Regina, his freedwoman and wife. Barates of Palmyra set this up. She was a Catuvellaunian by tribe, aged 30*”. Interestingly, after this relatively neutral description, Barates added a lament in his native language, Aramaic: “*Regina, the freedwoman of Barates, alas*!”. Barates changed languages probably because his emotional experience was best captured in his native tongue. Here, we address whether language context affects the development of a basic emotion, fear.

Emotions and feelings are at the core of human nature. We all experience joy, sadness, sorrow, contempt, and fear, and we often use language to communicate these feelings. Interestingly, the vehicle used to express these emotions seems to affect how we experience them. Highly emotional words or expressions do not seem to prompt the same emotional reaction in the native and foreign languages, as Barates’ story reveals^1,2^. Why this happens is still unknown, but it is probably related to how native and foreign languages are typically acquired. While native languages are acquired in emotionally rich family contexts, foreign languages are often learned in more emotionally neutral academic contexts^3,4^.

This differential emotional reactivity has widespread effects in various cognitive domains, as revealed by the so-called foreign language effect on decision-making and language processing. For example, loss and risk aversion are reduced in foreign language contexts^5,6^. Also, people tend to opt for utilitarian options more often in a foreign language when facing moral dilemmas^7^, and foreign language contexts affect automatic stages of emotional processing, modulating responses to self-related stimuli^8^. Finally, processing highly arousing negative sentences elicit a reduced psychophysical response when sentences are presented in a foreign language^9,10^. Three non-mutually exclusive factors have been suggested to be behind the foreign language effect: a reduction in emotional reactivity, a reduction in cognitive fluency, and an increase in psychological distance (see Costa *et al*.^11^ and Hayakawa *et al*.^12^, for reviews).We do not know, however, how these factors interact to give rise to this effect. In any case, the notion that foreign language contexts increase emotional distance and/or reduce emotional reactivity is receiving strong empirical support. This opens doors to the potential use of foreign languages in scenarios where it would be desirable a less (or more) emotional involvement, like conflict resolution, moral judgment, healthy choices, or even psychotherapy. Here we focus on whether fear acquisition can be modulated by the language context in which people are set.

The role of native vs. foreign language use in psychotherapy has a long history^13^. As already noted by Freud and colleagues, bilingual patients sometimes preferred using their second language when talking about anxiety related topics, a phenomenon called “the detachment effect”^14,15^. Similarly, Costa and Dewaele^16^ and Dewaele and Costa^17^ described how the use of a foreign language in psychotherapy allowed patients to talk about highly emotional material while feeling somewhat protected by this linguistic detachment. Thus, the use of a foreign language can promote certain level of psychological distance that can help people cope with emotional events. However, most of this evidence is somewhat anecdotic and comes mostly from clinical observations, lacking clear experimental evidence documenting this phenomenon. This is the first empirical study on the impact of using a foreign language for one important psychopathological mechanism, fear conditioning, which grounds one of the most effective psychotherapeutic techniques, exposure therapy. We asked a group of participants to complete a fear conditioning experiment in their native language, and another group completed it in their foreign language. This way, we explored whether a simple change in the linguistic context affects fear acquisition. If this were the case, not only would we better understand the mechanisms behind fear conditioning, but also foresee a new line of research and treatment where language context can help exposure therapy.

Fear conditioning is one the most important paradigms in psychology with clear clinical implications. The paradigm consists of presenting a neutral stimulus, the conditioned stimulus (CS), repeatedly in the presence of an aversive unconditioned stimulus (US), such as an electric shock. This pairing elicits a conditioned response of fear in the absence of the US, only presenting the CS. Fear acquisition and extinction were first conceptualized like low-level processes not involving cognitive processes, but this idea of mutual independence was rapidly discarded^18^. Fear can be acquired not only by direct learning (repeated pairing of CS-US), but also by observational learning (observing another person’s fear response to a neutral stimulus), and more importantly for our purposes, by verbal instruction (receiving verbal information about the aversive features of a neutral stimulus)^19^. Here we follow the work of Phelps *et al*.^20^, who employed an instructed fear paradigm where fear conditioning and the corresponding physiological response were elicited by just providing verbal instructions explaining the association between the CS and US without actually experiencing the US (see also Luck and Lipp^21^, for extensions and review). Hence, the use of verbal instructions for fear conditioning provides a unique opportunity to explore the extent to what different language contexts can modulate fear acquisition.

In our study, we adapted the experimental setting used by Phelps *et al*., and manipulated the language context of the experiment (namely, the language of the task: native or foreign). Verbal instructions were given either in the foreign or native language, and participants had to complete a series of countdowns either in one or the other language during the experiment. Skin conductance and pupil dilation data were collected as measures of the psychophysiological response to the conditioned and unconditioned stimuli. It is important to notice that psychophysiological responses are also affected by cognitive load, and that arguably foreign language processing is likely to increase such load. Indeed, other studies have demonstrated that foreign language contexts elicit larger pupil dilations than native language ones even in conditions with relatively low emotional connotation^10,22,23^. Hence, a correct comparison of the fear correlates across languages should consider the potential effect of cognitive load associated with foreign language processing. To do so, following the recommendations on human fear conditioning paradigms^24^, the current experiment follows a differential protocol including a neutral condition in which no fearful stimuli is expected (CS−), and in which participants have to perform the same task as in the fear condition (CS+). The critical issue then is whether the difference between the magnitude of the psychophysiological measures between the neutral and the fear conditions is larger in the native than in the foreign language, and not so much whether pupil dilation or skin conductance levels per se are larger in one language than in the other, since this may reflect just differences in the cognitive load. We predicted a different fear conditioning effect depending on the language context, being the difference between the CS+ and the CS− items lower in the foreign language.

Learning and conditioning have a key role in pathological emotion and behavior regulation, and concretely in the acquisition of fear, linked to important mental disorders like anxiety disorders and stress-related disorders. Thus, showing a mediating role of language context in processes related with fear conditioning can open doors to applications in psychotherapy.

## Results

Mean pupil size averaged across the right and the left eye (in an arbitrary measure provided by the eye-tracker) and mean electrodermal activity (in microSiemens, mS) were collected in two epochs for each trial. First, mean pupil size and electrodermal activity were averaged across the 4 seconds prior to each experimental trial, and served as the baseline activity. Second, the same measures were obtained for the whole period of 10 seconds corresponding to the participants’ countdown. Then, the mean percentages of change in the pupil dilation and in the skin conductance responses were computed for each participant by averaging the data of all the trials in each experimental condition with respect to the baseline epoch. Analyses were conducted with linear mixed-effect models (*lme*) using the *lme4*^25^ package in R^26^. Significance p-values and Type III F-statistics for main effects, interactions and planned comparisons were calculated using Satterthwaite approximations to denominator degrees of freedom as implemented in the *lmerTest* package^27^. For each dependent variable (Electrodermal Activity and Pupil Size) the fixed structure of the models was composed by the factors Language Context (native|foreign), Stimulus Type (threat|neutral, corresponding to the CS+ and CS− items, respectively), and the continuous variable Time (range: 1 to 10), as well as by their interactions. All models included maximal within-unit random effects structure^28,29^, thus random intercepts and random slopes for all the within-unit interaction terms were included. This lead to by participants random intercepts and random slopes for the interaction term of Stimulus Type and Time, and by trials random intercepts and random slopes for the interaction term of Stimulus Type, Language Context and Time. The predictor Time was centered to the mean prior to analysis so that the reference point in time for skin conductance and pupillary responses was set to the optimal mid value of the whole trial.

### Pupil Size

**Figure 1:**
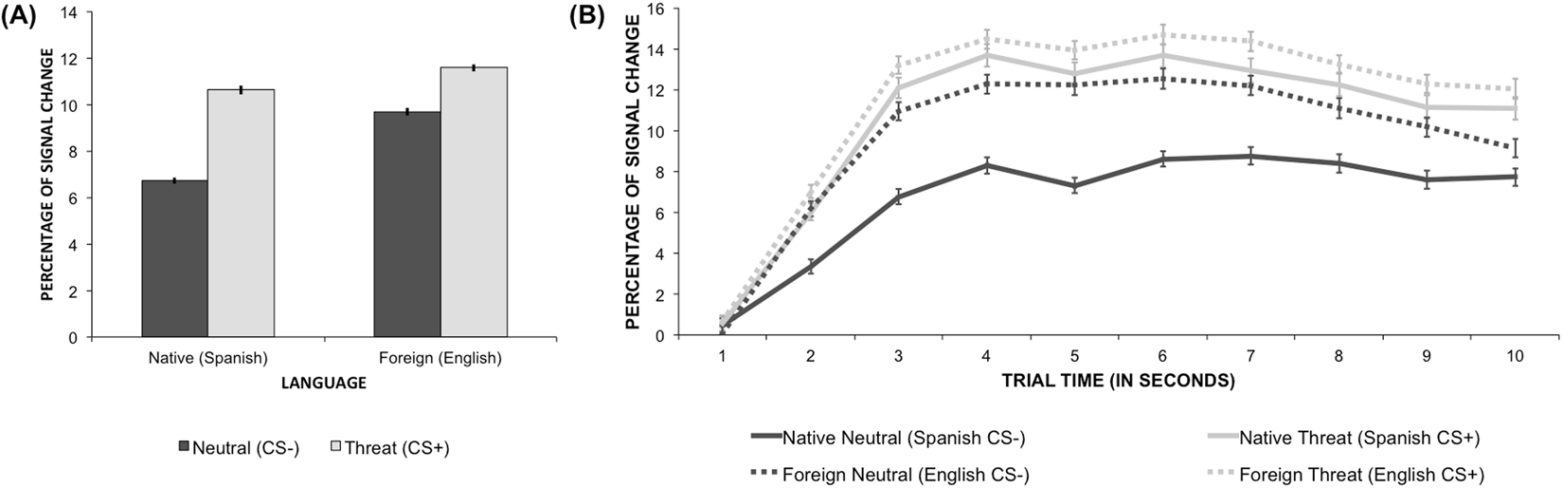
**(A)** Percentages of change in the pupil dilation with respect to the baseline epoch averaged by Stimulus Type and Language Context conditions. Light grey bars correspond to threat conditions (CS+) and dark grey bars correspond to neutral conditions (CS−). (**B**) Percentages of change of the pupillary responses averaged by Stimulus Type and Language Context conditions over the 10 time points corresponding to the 1-second bins of the trials. Light grey lines correspond to threat conditions (CS+) and dark grey lines correspond to neutral conditions (CS−). Solid lines correspond to native language conditions and dotted lines correspond to foreign language conditions. Error bars represent the standard error.

There was a main effect of Stimulus Type, showing that threat items resulted in significantly larger pupillary responses than neutral items [F(1,10481) = 471.17, p < .001, threat – neutral estimate = 3, lower and upper CI = 2.71: 3.25]. Trials in the foreign language resulted in significantly larger pupillary responses than trials in the native language as shown by the main effect of Language Context [F(1,52) = 4.11, p = 0.05, foreign – native estimate = 2, lower and upper CI = 0.02: 3.95]. Importantly, Stimulus Type interacted with Language Context [F(1, 10481) = 48.17, p < 0.001]. Planned comparison showed that the difference between threat and neutral trials was significant for both the native language [t(10481) = 20.27, p < 0.001] and the foreign language [t(10481) = 10.43, p < 0.001], and importantly, that this difference was twice as big for the former condition than for the latter [native language estimate (threat – native) = 3.9, lower and upper CI = 3.56: 4.32; foreign language estimate: 2, lower and upper CI = 1.65: 2.41; see Fig. 1A]. Pupillary responses significantly varied over time within the same trial [F(1,55.3) = 129.01, p < 0.001]. This change in pupillary responses over time within the same trial was in turn slightly modulated by Stimulus Type [F(1,50.5) = 3.13, p = 0.08]. That is, overall pupillary responses to threat trials tended to increase more than to neutral trials over time [Difference estimate (threat – neutral): Time 1 = 2.2 Time 10 = 3.64, increase: 1.44]. The rest of interactions were not found to approach significance (all p > 0.7; See Fig. 1B).

### Electrodermal Activity

**Figure 2:**
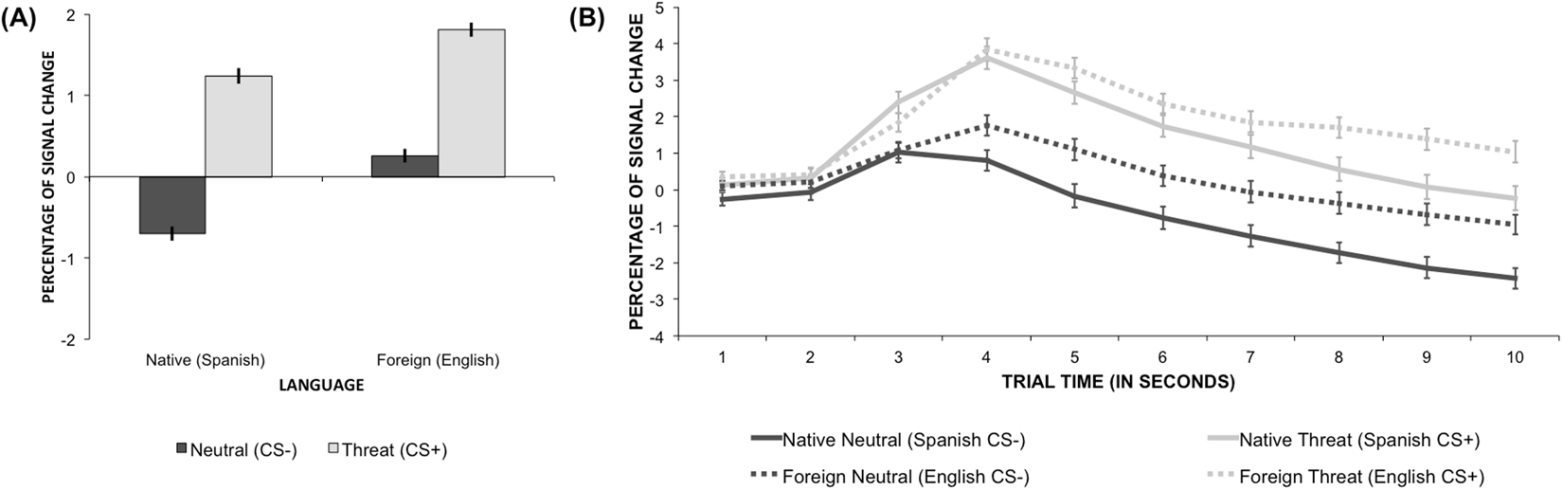
(**A**) Percentages of change in the electrodermal responses with respect to the baseline epoch averaged by Stimulus Type and Language Context conditions. Light grey bars correspond to threat conditions (CS+) and dark grey bars correspond to neutral conditions (CS−). (**B**) Percentages of change of the skin conductance averaged by Stimulus Type and Language Context conditions over the 10 time points corresponding to the 1-second bins of the trials. Light grey lines correspond to threat conditions (CS+) and dark grey lines correspond to neutral conditions (CS−). Solid lines correspond to native language conditions and dotted lines correspond to foreign language conditions. Error bars represent the standard error.

Threat items elicited larger skin conductance responses than neutral items, as shown by a significant main effect of Stimulus Type [F(1,10568) = 426.69, p < 0.001; threat – neutral estimate = 1.8, lower and upper CI = 1.59: 1.92]. The main effect of Language Context was also significant, showing that foreign language trials elicited higher levels of electrodermal activity than native language trials [F(1, 52) = 6.49, p = 0.01; foreign – native estimate = 0.8, lower and upper CI = 0.162: 1.36]. Importantly, Stimulus Type interacted with Language Context [F(1, 10570) = 5.32, p = 0.02; see Fig. 2A]. Planned comparisons showed that the difference in skin conductance responses between threat and neutral trials was significant for both the native language [t(10573) = 16.24, p < 0.001] and the foreign language context [t(10563) = 12.96, p < 0.001], and that this effect was larger for the former than for the latter conditions [native language (threat – native) estimate = 1.9, lower and upper CI = 1.71: 2.18; foreign language estimate = 1.6, lower and upper CI = 1.32: 1.79]. Skin conductance responses decreased over Time [F(1, 56.9) = 12.59, p < 0.001]. This decrease was in turn modulated by an interaction with Stimulus Type [F(1, 44.9) = 10.53, p = 0.002], showing that neutral items produced greater levels of reduction in electrodermal activity than threat items along the course of the trial, leading to increased differences between threat and neutral items over time [Difference estimate (threat – neutral): Time 1 = 0.84, Time 10 = 2.66, increase: 1.83; see Fig. 2B]. Skin conductance responses also tended to decrease more over time in native language conditions than in foreign language conditions [Difference estimate (foreign – native): Time 1 = 0.04, Time 10 = 1.49, increase: 1.46], although the interaction between Time and Language Context only approached significance [F(1, 55.9) = 3.44, p = 0.07]. The three-way interaction was not significant (p > 0.9).

## Discussion

We assessed the role of language context on fear conditioning, concretely on fear acquisition. Given that verbal instructions are enough to lead to fear conditioning, we argued that language contexts that reduce emotional reactivity might lead to a reduction on the intensity with which the neutral stimulus elicits the fear reaction. To test this hypothesis, we implemented an instructed fear experimental protocol in which participants were verbally informed that two different stimuli could be either cueing the potential presence of a threat stimulus or its absence. Most studies on human fear acquisition employ a differential protocol in which the CS+ is followed by the presence of the US, while a second one is not (CS−). The discrimination between the responses elicited by the CS+ and the CS− is the most critical statistical index of fear acquisition^24^. The instructed fear paradigm follows the same pattern, but participants are told beforehand the predicted value of the CS+ in comparison with the CS−^30^. As a measure of emotional reactivity, we registered participants’ changes in pupil size and electrodermal activity.

The stimulus associated with a threat stimulus led to a larger pupil size change than that associated with a neutral stimulus. This effect was robust and extended during the whole duration of the trials, and it reveals the power of verbal instructions to elicit a psychophysical response revealing fear, even when the threat stimulus is never actually presented. Importantly, and although the difference between the neutral and the threat conditions was present in both language contexts, its magnitude was larger in the native than in the foreign language context. It should be also noted that there was also a main effect of Language Context on the pupil dilation data, with pupillary responses being overall larger in the foreign language context than in the native language context. As discussed in the Introduction, this effect is likely due to the differences in the cognitive load associated with conducting a task in a foreign vs. in the native language (see also^10,22,23^). This main effect, however, does not compromise the interpretation of the difference between neutral and threat conditions as an index of fear conditioning.

The skin conductance results reinforce the pupil dilation data, offering a parallel interpretation and conclusion. Skin conductance changes for threat stimuli were greater than for neutral ones, and this difference was larger in the native language context. Thus, these results demonstrate that the two psychophysiological responses react to threat and neutral stimuli differently in a foreign than in a native language context, and that the two measures correctly capture the activity of the sympathetic nervous system in response to highly arousing stimuli^31^. These results reveal that language can induce fear conditioning, and more importantly, that the degree of permeability of the cognitive system varies as a function of the language context. The use of a native or a foreign language can modulate the strength with which verbal instructions yield an association between a neutral stimulus and a threat consequence.

These observations provide the first experimental evidence that certain verbal conditioning processes may be affected by language context. As we mentioned in the Introduction, the precise mechanisms driving foreign language effects are far from clear. We believe that the most plausible explanation of the differential effects of threat vs. neutral conditions in the native and foreign languages (namely, the fear conditioning effect) stems from a reduction on emotional reactivity being placed in a foreign language context. This reduction can help people to distance themselves from the situation, which in turn may diminish the fearful reaction that the CS+ may elicit. In fact, when the same data were explored by considering the time course of the threat conditioning effect in each language (from the first to the fourth quarter of the experiment) in order to ascertain whether fear conditioning extinguished faster in one language context than in the other, a similar conclusion was drawn. The conditioning effect in pupil size and electrodermal activity was relatively consistent thorough the experiment for the native language, but it showed a decreasing pattern in the foreign language, vanishing by the end of the experiment. Even if this finding results from an ad hoc reanalysis, it is indicative of fear conditioning being extinguished faster in the foreign than in the native language context, and this could align with the explanation based on the lower emotional reactivity of foreign languages. In summary, the main conclusion is that fear acquisition (namely, the CS+/CS−discrimination) is different in the native and in a foreign language, being more robust in the former than in the latter context. This interpretation fits well with the notion that foreign language processing can lead to a reduction of the contribution of heuristic intuitive processes driven by emotional reactivity on decision making^11,12^.

Our observations can also point to relevant practical issues in the context of clinical settings. For one thing, it is consistent with previous anecdotic observations regarding the so-called detachment effect in clinical practices discussed in the Introduction. But even more, given that the use of a foreign language impacts the processes behind fear conditioning, then it is possible that it may also affect fear extinction and return to fear. Fear conditioning is related to one of the most prevalent and impairing mental problems, anxiety disorders, reaching a lifetime prevalence of 28.8%^32^. Some of the key features of anxiety disorders may come about because neutral stimuli become aversive as a result of fear conditioning, impairing the life of patients who develop an irrational fear to neutral stimuli. Hence, studying fear acquisition and fear extinction is essential for the understanding and treatment of anxiety disorders^33,34^. Our study contributes to the understanding of the mediating role of language context in fear conditioning, opening doors to new developments in fear extinction and in the delivery of exposure therapy, a therapeutic strategy used for many of the anxiety disorders. Using a foreign language during exposure therapy could be an important modulator of emotional reactivity that may contribute to the effectiveness of this strategy.

To conclude, we report the first experimental evidence regarding how the use of a foreign language may reduce fear conditioning. The psychological distance elicited in foreign language contexts could be especially powerful in situations in which the actual threat stimulus is never presented. Future research needs to assess whether language context could indeed alter fear conditioning in situations in which the aversive stimulus is applied to the participants. The current findings open the avenue to the potential use of a foreign language on clinical contexts. Barates’ decision to express his sorrow for Regina’s death in his native language reveals how impactful language context can be when expressing our feelings and acquiring our fears.

## Methods

### Participants

After asking for participation in different classrooms, an initial sample of 203 undergraduate students from Universitat Jaume I showed interest in the study. The following inclusion criteria were set: age between 18 and 35 years old; Spanish as first language; have not studied in an English-speaking school; perceived level of knowledge in English between 6 and 9 (not perfect knowledge but still relatively proficient); and have lived less than 12 months in an English-speaking country. A large number of students from the initial sample did not fulfill the inclusion criteria or refused to participate, and the experiment was finally carried out with 54 students who met the criteria, attended and completed the experiment.

All the final sample of participants had normal or corrected-to-normal vision, they were right-handed and they were neurologically intact native Spanish speakers who were not consuming any psychopharmaceutic drug. The group of 27 participants (18 females) randomly assigned to the foreign language experimental context and the group of 27 participants (18 females) assigned to the native language context were matched for a number of sociodemographic, cognitive and linguistic factors (see Table 1). To this end, participants completed a short sociodemographic questionnaire and a questionnaire aimed at assessing their English skills, knowledge and use. Besides, participants also completed a 6-minutes abridged version of the Kaufman Brief Intelligence Test^35^ (K-BIT), the Empathy Quotient questionnaire^36^ (EQ), and the State-Trait Anxiety Inventory^37^ (STAI). Participants in the two language context groups did not differ in any of these variables, and the number of participants per group and condition was in or above the range reported in earlier studies^20^, so that sufficient statistical power could be granted.

**Table 1:** Characteristics of the two samples of participants assigned either to the native or to foreign language context. Each mean is followed by the corresponding standard deviation within parentheses.

	Language Context	
	Native (n = 27)	Foreign (n = 27)
Females (number)	18	18
Age (in years)	22.93 (3.91)	22.44 (3.57)
Persons at home	3.30 (1.07)	3.35 (1.09)
Monthly personal income (in euros)	435 (658)	309 (517)
Monthly family income (in euros)	2224 (1142)	2156 (1176)
Self-perceived general English proficiency (1-to-10 scale)	7.07 (0.87)	6.89 (1.31)
Self-perceived verbal comprehension English proficiency (1-to-10 scale)	7.70 (1.56)	7.19 (1.42)
Self-perceived written comprehension English proficiency (1-to-10 scale)	8.22 (0.85)	8.11 (1.12)
Self-perceived verbal production English proficiency (1-to-10 scale)	6.78 (1.22)	6.81 (1.44)
Self-perceived written production English proficiency (1-to-10 scale)	7.33 (1.24)	7.33 (1.24)
Exposure to English (%)	33.41 (21.94)	35.93 (21.31)
Age of English acquisition (in years)	7.89 (4.46)	7.48 (3.32)
STAI – State (score)	15.41 (5.70)	15.19 (6.71)
STAI – Trait (score)	19.37 (8.35)	17.44 (8.05)
Empathy Quotient (score)	43.33 (9.70)	43.22 (10.36)
IQ (correct responses)	21.26 (3.64)	20.78 (3.19)

### Design

We employed an instructed fear paradigm with a differential protocol, where participants were instructed that a stimulus (CS+) would be followed by a US and another stimulus (CS−) would not^24,30^. A 2*2 design was followed with Stimulus Type (threat|neutral) set as a within-participant factor and Language Context (native|foreign) set as a between-participants factor. Hence, four experimental conditions were established for the instructed fear paradigm according to Language Context and Stimulus Type: foreign + threat; foreign + neutral; native + threat; native + neutral. Participants were randomly assigned to one of the two levels defined by the Language Context factor. The levels of Stimulus Type (threat|neutral) were associated to the specific color of the depicted geometric shapes, which could be either blue or yellow. The level-color association was counterbalanced across participants.

### Dependent Variables

During the experimental phase, two critical dependent measures were gathered. First, the pupils’ diameter in response to each stimulus was collected using an eye-tracker (Tobii® TX300), given that the pupil diameter is a good indicator of emotion charge regarding aversive stimuli^38^. Second, electrodermal activity was also registered in order to allow for an analysis of the skin conductance in response to the critical stimuli, given that this is a key indicator of automatic emotion responses^20,39^. To this end, electrodermal information was collected using a Biopac® EDA-100C device.

### Procedure

The study was approved by the Universitat Jaume I Ethics Committee. All participants were volunteers and gave their informed consent prior to the data collection. The experiment was performed in accordance with relevant national and international guidelines and recommendations. Researchers asked for permission to professors in university degrees related to the study of languages: Philology and Translation studies. We chose these degrees in order to increase the chance of finding participants with some competence in English as a second language. Once the permission was granted, one of the researchers publicized the experiment in two degrees (Philology and Translation studies) without revealing the specific goal of the study. Students who were interested filled out a brief questionnaire that explored the most relevant inclusion criteria and provided their contact details. Students who fulfilled the inclusion criteria were then contacted and an appointment was set for them. The experiment was conducted individually at Laboratorio de Psicologia y Tecnologia (Lapsitec) at Universitat Jaume I in two cabins. In a first room one of the researchers (researcher 1) welcomed the participant and asked her to fill out some questionnaires on the computer and to read and sign the consent form. After this, the participant was randomly assigned to one experimental group (i.e., native or foreign language context).

After this, the participant was guided by researcher 1 to the room where the experimental phase was conducted. Two researchers (researchers 2 and 3) were in charge of this phase. Researcher 2 was the one interacting with the participant either in Spanish or English depending of the experimental group. Researcher 3 was in charge of attaching the different sensors and electrodes and calibrating the eye-tracker. Researcher 3 first attached the sensors to collect the electrodermal activity while researcher 2 explained the goal of these sensors. After correctly placing the sensors to monitor skin conductance, the participant completed the 6-minutes abridged version of the IQ test whose aim was twofold. First, this gave a rough estimate of the participant’s intellectual abilities to allow for inter-group matching (see Participants section). And second, this allowed for a sufficient temporal frame to stabilize the electrodermal activity.

After finishing the IQ test, the participant was presented with the critical instructed fear task following the experimental paradigm from Phelps *et al*.^20^. Researcher 3 calibrated the eye tracker and attached an electrode to the participant’s wrist while Researcher 2 explained that this was set for the administration of the electric shock. Researcher 2 told the participant that her eye movements would be monitored during the task and that there would be the possibility of receiving between 1 and 3 mild electric shocks during the task. Although none electric shock was delivered, participants were told that this would happen in order to achieve fear conditioning. The participant was informed that she would see a series of trials consisting on blue or yellow squares presented on the center of the screen with a number superimposed to them, changing from 10 to 1 in a sequential descendent order (a countdown) at a pace of 1 second per number. The task of the participant was to say out loud the numbers (in Spanish or English depending on the experimental condition). Importantly, the participant was also told that electric shocks would be only delivered when the squares were in one specific color (blue or yellow, depending on the counterbalanced experimental condition). That is, there were threat and safe trials depending on the color of the square. After reading the instructions, researcher 2 made sure that the participant had fully understood the procedure and commands prior to starting the task. During the experiment, 10 trials were included for each condition (threat vs safe) and each trial lasted for 10 seconds. The order of the trials was randomized for each participant. During the experimental phase, all the interactions with the participant were performed in the language context assigned (Spanish or English). This included all the task instructions (written and spoken), the questions raised by the participant, the answers given by the experimenters, and more importantly, the 10-number countdown in each of the trials. This phase lasted for about 25 minutes.

Right at the end of the experiment, researcher 1 took the participant to the first room and debriefed her letting her know that she was in a non-shock condition. The full experimental session lasted for around 45 minutes.

### Data availability

The datasets generated during and/or analysed during the current study are available from the corresponding author on reasonable request.
