# Author Correction: The effect of foreign language in fear acquisition

**DOI:** 10.1038/s41598-018-24106-7

**Published:** 2018-04-13

**Authors:** Azucena García-Palacios, Albert Costa, Diana Castilla, Eva del Río, Aina Casaponsa, Jon Andoni Duñabeitia

**Affiliations:** 1 Department of Basic and Clinical Psychology and Psychobiology, Jaume I University, Castellón, Spain; 2 Ciber. Fisiopatología Obesidad y Nutrición, (CIBERObn), Instituto de Salud Carlos III, Madrid, Spain; 3 Center for Brain and Cognition, Universitat Pompeu Fabra, Barcelona, Spain; 4 Institució Catalana de Recerca i Estudis Avançats (ICREA), Barcelona, Spain; 5 Department of Linguistics and English Language, Lancaster University, Lancaster, United Kingdom; 6 Facultad de Lenguas y Educación, Universidad Nebrija, Madrid, Spain; 7 Basque Center on Cognition, Brain and Language (BCBL), Donostia, Spain

Correction to: *Scientific Reports* 10.1038/s41598-018-19352-8, published online 18 January 2018

The Acknowledgements section in this Article is incomplete.

“We would like to thank Dr. Miguel F. Ruiz-Garrido and his colleagues from The Department of English Studies at Jaume I University and Irma Mulet and her colleagues of the Language and Terminology Service at Jaume I University for providing access to the students that participated in this study. We would also like to thank all the participants for their valuable collaboration. This research has been partially funded by grants PSI2015-65689-P and SEV-2015-0490 from the Spanish Government and AThEME-613465 from the European Union.”

should read:

“We would like to thank Dr. Miguel F. Ruiz-Garrido and his colleagues from The Department of English Studies at Jaume I University and Irma Mulet and her colleagues of the Language and Terminology Service at Jaume I University for providing access to the students that participated in this study. We would also like to thank all the participants for their valuable collaboration. This research has been partially funded by grants PSI2015-65689-P, PSI2014-52181-P and SEV-2015-0490 from the Spanish Government and AThEME-613465 from the European Union.”

